# Reinforcement History Dependent Effects of Low Dose Ethanol on Reward Motivation in Male and Female Mice

**DOI:** 10.3389/fnbeh.2022.875890

**Published:** 2022-04-11

**Authors:** Kathleen G. Bryant, Binay Singh, Jacqueline M. Barker

**Affiliations:** Barker Lab, Department of Pharmacology and Physiology, Drexel University College of Medicine, Philadelphia, PA, United States

**Keywords:** sex, reward, motivation, microstructure, ethanol, schedule

## Abstract

Alcohol use disorders (AUDs) are more prevalent in men than in women, though AUD diagnoses in women are growing rapidly, making an understanding of sex differences in alcohol-related behaviors increasingly important. The development of AUDs involves the transition from casual, low levels of alcohol drinking to higher, maladaptive levels. The ability of low dose alcohol to drive reward and drug seeking may differ in males and females, and this could underlie differences in susceptibility to AUD. In this study we sought to determine whether a history of chronic, low dose ethanol exposure (0.5 g/kg; i.p.) could drive sucrose reward seeking and motivation, and whether this differed between male and female mice. Adult mice were trained to lever press for a liquid sucrose reward on two reinforcement schedules: a random interval (RI) schedule and a variable ratio (VR) schedule. After training, mice were tested on each of these levers for reward motivation using a progressive ratio test. We found that a history of low dose ethanol exposure increased sucrose reward motivation in male mice, but only on the RI lever and only when exposure occurred proximal to learning. Female mice were more motivated for sucrose on the RI lever than the VR lever regardless of ethanol exposure condition. These findings indicate that training on different reinforcement schedules affects reward motivation. Further, we show that males are more susceptible to the effects of low dose ethanol on sucrose reward motivation than females. These data broaden our understanding of sex differences in reward seeking as a result of ethanol exposure.

## Introduction

While alcohol use disorders (AUDs) present a significant societal and economic burden, the majority of people who drink alcohol do so at low, casual levels that do not reach criteria for an AUD (SAMHSA, 2019). However, these chronic, lower drinking levels can produce behavioral and neurobiological changes that may promote the transition from casual drinking to heavy drinking seen in the development of AUD. A greater understanding of how low levels of alcohol drinking or alcohol exposure impact inflexible behavior can increase understanding of how susceptibility to AUD is conveyed.

The physiological effects of acute ethanol are distinct at low doses vs. high doses. Lower doses (0–0.75 g/kg) produce stimulatory effects, while higher doses (1 g/kg +) produce sedative effects (Cui and Koob, 2017). Low dose ethanol has also been shown to be more neuroprotective, as it reduces inflammation and increases production of neurotrophic factors (Gahring et al., 1999; Tizabi et al., 2018). However, low dose ethanol effects on reward-seeking behavior are neither well known nor well studied. Clinical and preclinical studies have shown that the impacts of ethanol exposure on memory and behavior depend on the timing of exposure in relation to learning (Tyson and Schirmuly, 1994). It has also been shown that acute ethanol exposure, even at low doses, up to 2 h after a learning event can promote memory recall (Alkana and Parker, 1979; Hewitt et al., 1996). Further supporting the importance of exposure timing, conflicting results have been found when chronic ethanol exposure occurs proximal to learning vs. distal (Corbit et al., 2012; Barker et al., 2020).

Men are currently more likely to be diagnosed with an AUD than women, but the gap has been narrowing in recent years (Keyes et al., 2008; White, 2020). It is especially important to understand how sex may impact low dose alcohol effects as women escalate from casual use to addiction more rapidly than men and may therefore be differentially susceptible to low dose ethanol effects (Becker and Koob, 2016). Further, women suffer greater negative health outcomes with more rapid onset and from lower doses of alcohol than men (Foster et al., 2018), so it is crucial to investigate outcomes of low dose ethanol in both sexes to understand the course of alterations.

As research into sex differences progresses, it is increasingly clear that male and female rodents exhibit differing patterns of reward seeking. Generally, female rodents have been found to be more motivated than males to work for highly palatable foods, like sucrose (Seaman et al., 2008; Sherrill et al., 2011; Sinclair et al., 2017). Females also develop sucrose-seeking habits faster than males (Quinn et al., 2007). There appear to be no striking sex differences in the attribution of incentive salience to food cues (Pitchers et al., 2015), suggesting that increased motivation observed in females is not driven by a greater sensitivity to food-related cues. In addition to these baseline differences, exposure to drugs and alcohol may have sex-specific or sex-determined outcomes. For example, it has been shown that the effects of higher doses of ethanol on behavior can depend on sex and, in some cases, even reinforcement schedule (Chaudhri et al., 2005; Barker et al., 2017a; Giacometti et al., 2020). Despite this, there is a lack of research into sex differences in the outcomes of lower doses of ethanol.

This study thus investigated the effects of low dose ethanol exposure on male and female mice to determine whether repeated low dose ethanol exposure impacted sucrose reward seeking and motivation. Further, we investigated how timing of low dose ethanol exposure and training history impacted outcomes.

## Materials and Methods

### Subjects

Adult male and female C57BL/6J mice (9 weeks of age; 42 males, 30 females) from The Jackson Laboratory were used in these studies in accordance with the Drexel University Institutional Animal Care and Use Committee guidelines. The mice were housed in a vivarium with a standard 12:12 h light/dark cycle and were given 1 week to acclimate to the facility before beginning any experiments. Some mice (24 males and 18 females) underwent stereotaxic surgery with a pAAV-hSyn-EGFP retrograde adeno-associated virus (Addgene plasmid # 50465, RRID: Addgene_50465) targeting the nucleus accumbens shell (AP + 1.5 mm ML + 0.6 mm DV −4.7 mm) prior to beginning behavioral experiments. These mice were not used for any other experiments. Following recovery from surgery or following the acclimation period, mice were restricted to approximately 90% of their *ad libitum* weight and were then maintained at that weight for the length of the experiments. All mice were group housed for the duration of the study.

### Operant Set-Up

All operant training occurred in standard Med-Associates operant boxes for mice, housed within sound attenuating chambers that included a fan for ventilation and white noise. The left wall of the box was curved and featured five nose poke holes with lights that were not activated or used for any of these studies. The right wall of the chamber had two retractable levers on either side of a reward magazine that had slots for pellet and/or liquid reinforcer. A house light was fitted above the magazine. The back wall, door, and ceiling of the box were made with Plexiglas. The floor was made with standard metal bars and was raised above a removable tray. Besides the house light, which turned on at the start of the session and remained on for the length of the session, there were no discrete cues presented during any of the behavioral sessions.

### Instrumental Training

Prior to starting instrumental training, mice were habituated to the operant box and reward delivery magazine. For these sessions, the mice were placed in the operant box and the 10% liquid sucrose reward (20 ul, in tap water) was delivered into the magazine every 60 s for a total of 15 min. Mice only had one magazine training session per day. After 2 days of magazine training, mice were trained to lever press for sucrose on two separate levers. Only one lever was accessible at a time, and the levers were presented consecutively during the session. Thus, for the first half of the session the mice had access to one lever, then that lever would retract, and they would have access to the other lever for the rest of the session. Each lever was accessible for 15 min, and the whole session lasted 30 min. The order of which lever was accessible first alternated each day for each mouse and was counterbalanced across all groups and conditions.

Initially, both levers delivered reinforcer on a fixed ratio 1 (FR1) schedule where each lever press resulted in reward delivery. Mice were trained on the FR1 schedule until they reached stable responding (at least 15 lever presses on each lever, maintained for 3 days). Mice that did not reach stable responding on both levers were excluded (seven males, one female). The schedules of reinforcement for each lever then diverged, such that the left lever began reinforcing on a random interval (RI) schedule and the right lever began reinforcing on a variable ratio (VR) schedule. On a RI schedule, the first press after a randomly determined interval (averaging 30 s for RI30, and 60 s for RI60) has elapsed was reinforced. On a VR schedule, the first lever press after a variable number of presses was reinforced (averaging 5 presses for VR5, and 8 presses for VR8). It has been shown that RI schedules promote inflexible behavior whereas VR schedules maintain flexible behavior (Adams and Dickinson, 1981; Dickinson et al., 1983; Gremel and Costa, 2013; Barker et al., 2017a). Mice were trained for 3 days on the RI30/VR5 schedule and then for 3 days on the RI60/VR8. After training, a subset of mice was tested for inflexible behavior on a contingency degradation and outcome devaluation test before beginning testing on the progressive ratio (PR) schedule. Data from those additional tests are being excluded for the purposes of this manuscript.

### Ethanol Exposure

Previous studies have shown that the effects of ethanol on learning can depend on exposure timing in relation to behavior, especially if exposure occurs within protein synthesis dependent memory consolidation (e.g., 1–3 h after learning) (Bourtchouladze et al., 1998; Hernandez and Abel, 2008). This study tested and controlled for exposure timing dependent effects by injecting saline or low dose ethanol (0.5 g/kg; i.p.) daily either 1 h (during this window) or 4 h (outside of this window) after behavior. No differences were observed between saline mice that received injections at 1 vs. 4 h, so saline mice were collapsed across groups. Mice were exposed to ethanol starting on the first day of FR1 training through the last day of RI60/VR8 training. There was no further ethanol exposure after the last day of training, therefore there was no ethanol exposure proximal to the PR testing. PR testing took place 1–3 weeks after the final training day (i.e., after last ethanol exposure). The time between the final ethanol exposure and PR testing was determined by training and acquisition length, and there were no differences across groups and sexes.

### Progressive Ratio Testing

As PR testing began 1–3 weeks after the final RI60/VR8 training session, mice were given two additional days of RI60/VR8 retraining before beginning testing on the PR. There was no additional ethanol exposure on these retraining days. Mice were tested for sucrose reward motivation on the PR test, which measures how much an animal is willing to lever press for a particular reward. The PR schedule used here was an arithmetic schedule, where the number of lever presses required for reinforcer delivery increased by 4 every time reward was delivered. An arithmetic schedule was chosen as this has been shown to be sufficient for measuring appetitive motivation in rodents previously (Gourley et al., 2008). The test session ended either after 5 min had passed without a lever press (e.g., the “breakpoint”) or when the maximum session length was reached. The maximum session length was 4 h for the first cohort of mice (*n* = 20 males) but was changed to 8 h for all subsequent cohorts when four mice hit the maximum session length without reaching their breakpoint. Most mice reached their breakpoints within the confines of the session length, regardless of whether it was 4 or 8 h. For the RI lever, four males and one female did not reach their breakpoint; for the VR lever two males did not reach their breakpoint. Mice that did not reach their breakpoints were still included in the analysis using the maximum breakpoint reached at session termination. There were no differences between mice that had the 4 h capped sessions vs. the 8 h capped sessions. Each mouse was only tested once for each lever on each day, and the order of which lever was tested first was counterbalanced across all groups and conditions.

It is important to understand not just the whole behavioral output, but also the differences that exist as part of a “behavioral microstructure.” Investigating this microstructure can reveal latent differences in behavioral strategy that are not otherwise clear using traditional measures (Robinson and McCool, 2015; Fuchs et al., 2019; Yamada and Kanemura, 2020). For example, differences may exist in how often a mouse checks the reward magazine for reward delivery, which may reflect reward tracking or attention to reinforcement schedule. Differences in these strategies between mice and between groups may reflect different mechanisms, even if the overall behavioral output or phenotype is the same. Thus, one measure of interest for this study was magazine checking after a lever press, as differences in reward delivery tracking, as measured by what percentage of lever presses were followed by a magazine entry, could relate to reward evaluation. Alternatively, magazine checking could relate to sensitivity to the PR, as a mouse that checks the magazine more often for reward may also be more sensitive to progressively increasing lever press requirements on the PR and would stop responding sooner.

### Statistical Analyses

GraphPad PRISM was used for all statistical analyses. A repeated measures ANOVA (rmANOVA) or mixed effects analysis (when there were missing values) was used for all training and testing data. Sidak’s, Tukey’s, and Dunnett’s corrections were used for *post-hoc* analyses as appropriate. Correlational analyses were performed using linear regression.

## Results

### Effects of Low Dose Ethanol and Schedule on Sucrose Reward Seeking in Males

**FIGURE 1:**
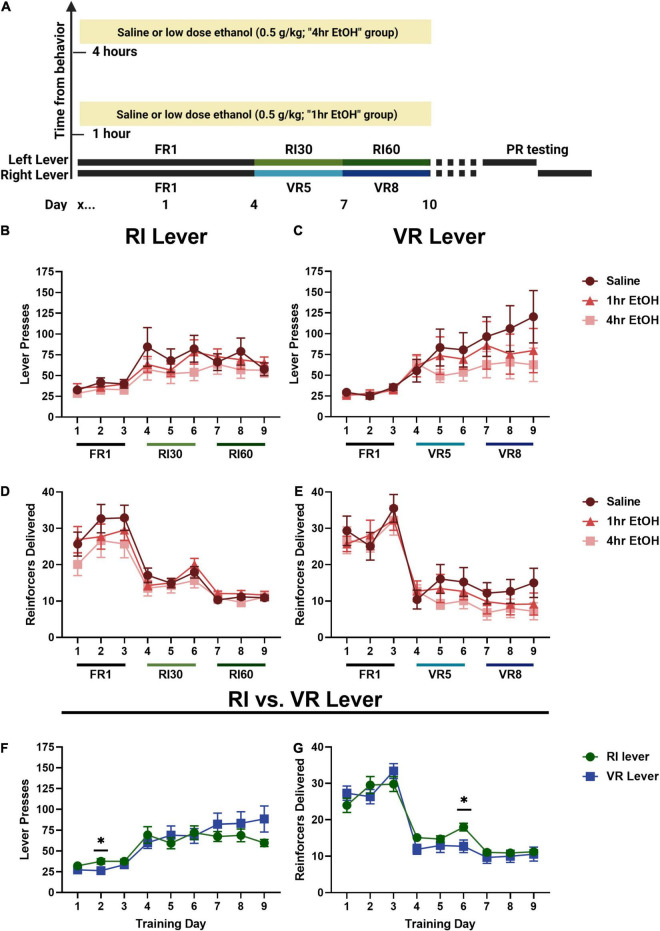
Low dose ethanol exposure does not impact basal reward seeking in males. **(A)** A timeline of the behavioral experiments and ethanol exposure. In males, there is no effect of low dose ethanol exposure on responding during training on the RI **(B)** or VR **(C)** lever. There is also no effect of low dose ethanol exposure on reinforcer delivery during training on the RI **(D)** or VR **(E)** lever. Overall, neither lever pressing **(F)** nor reinforcer delivery **(G)** was different on the RI or VR lever for males. Data shown as mean ± SEM (*p < 0.05).

To determine whether low dose ethanol impacted reward seeking behavior, adult male and female mice were trained to lever press for sucrose on two levers with differing reinforcement schedules (Figure 1A). In males, a main effect of day was observed on the RI lever (Figure 1B) [rmANOVA, *F*_(2.787, 89.20)_ = 13.45, *p* < 0.0001], with *post-hoc* analyses revealing a significant escalation in responding on the last day of training as compared to the first (Sidak’s, *p* < 0.0001). No main effect of ethanol exposure [*F*_(2, 32)_ = 0.6281, *p* = 0.5401] nor interaction [day × exposure, *F*_(16, 256)_ = 0.7632, *p* = 0.7264] was observed on the RI lever. A main effect of day was also observed on the VR lever (Figure 1C) [rmANOVA, *F*_(1.796, 55.69)_ = 13.50, *p* < 0.0001], with *post-hoc* analyses revealing a significant escalation in responding on the last day of training as compared to the first (Sidak’s, *p* = 0.0020). Similar to the RI lever, no main effect of ethanol exposure [*F*_(2, 31)_ = 0.6349, *p* = 0.5368] or interaction [day × exposure, *F*_(16, 248)_ = 1.099, *p* = 0.3562] was observed on the VR lever. These findings show that responding escalated on both RI and VR schedules, and that post-training, low dose ethanol exposure did not impact basal reward seeking on these schedules in males.

Differences in the number of reinforcers delivered across training were also examined in males. On the RI lever (Figure 1D), a main effect of day was observed [rmANOVA, *F*_(3.530, 112.9)_ = 47.81, *p* < 0.0001]. *Post-hoc* analyses revealed that the number of reinforcers delivered on the last day of training was significantly reduced as compared with the first day (Sidak’s, *p* < 0.0001), consistent with the leaner reinforcement schedule. A main effect of day was also observed for reinforcer delivery on the VR lever (Figure 1E) [rmANOVA, *F*_(4.343, 134.6)_ = 71.49, *p* < 0.0001] with *post-hoc* analyses revealing that the number of reinforcers delivered on the last day of training was significantly reduced as compared with the first day (Sidak’s, *p* < 0.0001).

As there were no effects of ethanol exposure on lever pressing on either lever, male mice were collapsed across exposure conditions to compare responding on the two levers directly (Figure 1F). A main effect of training day [Mixed effects analysis, *F*_(1.734, 58.95)_ = 21.71, *p* < 0.0001] and a day × lever interaction [*F*_(2.568, 84.43)_ = 3.533, *p* = 0.0236] were observed; Sidak’s *post-hoc* analysis showed that lever pressing was significantly higher on the second day of FR1 training on the RI lever as compared to the VR lever in males (*p* = 0.0284). Male reinforcer delivery data were collapsed across exposure conditions to compare overall reinforcer delivery on the RI vs. VR lever (Figure 1G). A main effect of training day [rmANOVA, *F*_(4.518, 149.1)_ = 134.9, *p* < 0.0001] and a day × lever interaction [*F*_(3.926, 129.6)_ = 3.180, *p* = 0.0164] were observed; Sidak’s *post-hoc* analysis showed that male mice received significantly more sucrose reinforcers on the RI lever as compared to the VR lever on the 3rd day of RI30/VR5 training (day 6 overall; *p* = 0.0248).

### Effects of Low Dose Ethanol and Schedule on Sucrose Reward Seeking in Females

**FIGURE 2:**
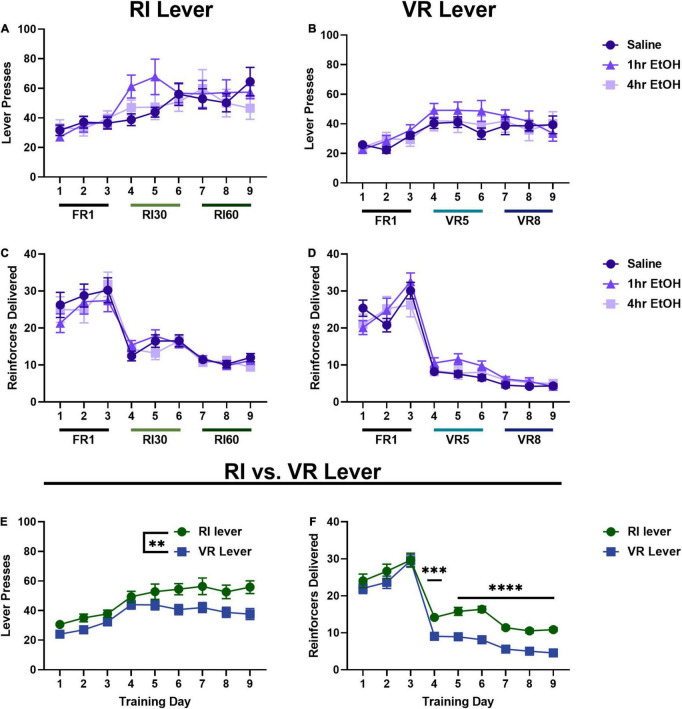
Reinforcer schedule but not ethanol exposure impacts reward seeking in females. In females, there is also no effect of low dose ethanol exposure on responding on the RI **(A)** or VR **(B)** lever. There is also no effect of low dose ethanol exposure on reinforcer delivery on the RI **(C)** or VR **(D)** lever. Overall, females press more **(E)** and receive more reinforcers **(F)** on the RI lever as compared to the VR. Data shown as mean ± SEM (**p < 0.01, ***p < 0.001, and ****p < 0.0001).

In females, when comparing lever presses across exposure conditions on the RI lever (Figure 2A) a main effect of day was observed [rmANOVA, *F*_(5.035, 129.0)_ = 10.42, *p* < 0.0001], with *post-hoc* analyses revealing a significant escalation in responding on the last day of training as compared to the first day (Sidak’s, *p* < 0.0001). No main effect of ethanol exposure [*F*_(2, 26)_ = 0.3853, *p* = 0.6841] nor interaction [day × exposure, *F*_(16, 205)_ = 1.446, *p* = 0.1234] were observed on the RI lever. On the VR lever (Figure 2B), a main effect of day was also observed [rmANOVA, *F*_(5.629, 144.2)_ = 9.456, *p* < 0.0001], with *post-hoc* analyses revealing a significant escalation in responding on the last day of training as compared to the first (Sidak’s, *p* = 0.0034). No main effect of ethanol exposure [*F*_(2, 26)_ = 0.4340, *p* = 0.6525] or interaction [day × exposure, *F*_(16, 205)_ = 0.8284, *p* = 0.6524] were observed on the VR lever. Similar to data from the males, these findings show a significant escalation of responding on RI and VR schedules, but no effect of post-training, low dose ethanol on basal reward seeking in females.

Reinforcer delivery across training was also analyzed in females. On the RI lever (Figure 2C), a main effect of day was observed [rmANOVA, *F*_(3.667, 94.88)_ = 46.89, *p* < 0.0001] with *post-hoc* analyses revealing reinforcer delivery was significantly reduced on the final day of training as compared to the first day (Sidak’s, *p* < 0.0001). A main effect of day was also observed on the VR lever (Figure 2D) [rmANOVA, *F*_(3.764, 93.64)_ = 122.0, *p* < 0.0001], with *post-hoc* analyses showing that reinforcer delivery was significantly reduced on the final day of training as compared to the first day (Sidak’s, *p* < 0.0001). As in males, these reductions in total reinforcer delivery are consistent with the increasingly lean reinforcement schedules.

As there were no effects of ethanol exposure on lever pressing on either lever, female mice were collapsed across exposure conditions to compare responding on the two levers directly (Figure 2E). A main effect of training day [Mixed effects analysis, *F*_(4.768, 133.5)_ = 17.90, *p* < 0.0001] and lever [*F*_(1.000, 28.00)_ = 9.453, *p* = 0.0047] were observed, with female mice pressing significantly more on the RI lever as compared to the VR lever. Female reinforcer delivery data were also collapsed across exposure conditions to compare overall reinforcer delivery on the RI vs. VR lever (Figure 2F). A main effect of training day [Mixed effects analysis, *F*_(5.005, 135.1)_ = 139.8, *p* < 0.0001], lever [*F*_(1.000, 27.00)_ = 28.12, *p* < 0.0001], and a day × lever interaction [*F*_(2.981, 79.74)_ = 3.332, *p* = 0.0238] were observed. Sidak’s *post-hoc* analysis showed that reinforcer delivery was significantly higher on the RI lever than the VR lever for all RI30/VR5 and RI60/VR8 training (training days 4–9; day 4 *p* = 0.0004, days 5–9 *p* < 0.0001).

### Effects of Low Dose Ethanol on Sucrose Reward Motivation

**FIGURE 3:**
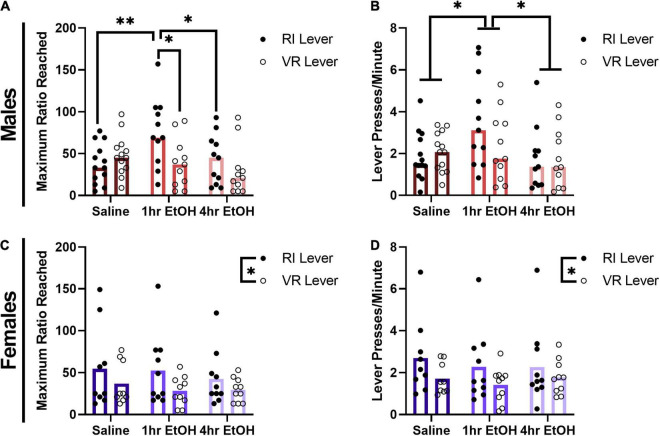
Effect of low dose ethanol on sucrose reward motivation depends on sex and reinforcement schedule. **(A)** Male mice with a history of 1 h EtOH exposure are significantly more motivated for sucrose, but only on the RI lever. **(B)** One hour EtOH male mice also exhibit significantly higher response rates during the PR. **(C)** Female mice are more motivated for sucrose on the RI lever, regardless of exposure history. **(D)** Female mice also exhibit higher response rates on the RI lever during PR testing. Data shown as mean ± SEM (*p < 0.05, **p < 0.01).

To determine whether a history of low dose ethanol impacted sucrose reward motivation, male and female mice were tested on a PR schedule on the levers previously reinforced on RI or VR schedules. In males, a significant exposure condition × lever interaction was observed for the maximum ratio reached during the PR session (Figure 3A) [rmANOVA, *F*_(2, 32)_ = 3.492, *p* = 0.0425]. *Post-hoc* analyses revealed that male mice with a history of exposure occurring proximal to learning (1 h EtOH group) reached significantly higher ratios on the RI lever vs. the VR lever (Sidak’s, *p* = 0.0241). Further, 1 h EtOH male mice also reached higher ratios on the RI lever as compared to saline- (Tukey’s, *p* = 0.0075) and 4 h EtOH-exposed (Tukey’s, *p* = 0.0298) mice. A three-way ANOVA revealed that there were no effects of surgical history on breakpoints [surgery, *F*_(1, 29)_ = 0.5864, *p* = 0.4500; ethanol × surgery × lever, *F*_(2, 29)_ = 1.306, *p* = 0.2863], and thus animals were collapsed across history of surgery for further analyses. When comparing overall response rates on the PR (Figure 3B), a main effect of exposure condition was observed [rmANOVA, *F*_(2, 32)_ = 4.402, *p* = 0.0205]. *Post-hoc* analyses indicated that 1 h EtOH male mice had significantly higher response rates as compared to saline- (Dunnett’s, *p* = 0.0456) and 4 h EtOH-exposed (Tukey’s, *p* = 0.0248) mice. These results suggest that the effects of low dose ethanol on reward motivation are determined by not only exposure timing, but also reinforcement schedule history in male mice.

In female mice, for the maximum ratio reached within the PR session a main effect of lever was observed (Figure 3C) [rmANOVA, *F*_(1, 26)_ = 6.264, *p* = 0.0189] such that female mice reached significantly higher ratios on the RI lever as compared to the VR lever. There was no main effect of exposure condition [*F*_(2, 26)_ = 0.3414, *p* = 0.7139] or exposure condition × lever interaction [*F*_(2, 26)_ = 0.2013, *p* = 0.8189] observed in females for the maximum ratio reached. A three-way ANOVA revealed that there were no effects of surgical history on breakpoints [surgery, *F*_(1, 23)_ = 3.064, *p* = 0.0934; ethanol × surgery × lever, *F*_(2, 23)_ = 0.1006, *p* = 0.9047], and thus animals were collapsed across history of surgery for further analyses. A similar pattern was seen when comparing response rates on the PR test in females (Figure 3D), where there was again a main effect of lever [rmANOVA, *F*_(1, 26)_ = 5.877, *p* = 0.0226]. No main effect of exposure condition [*F*_(2, 26)_ = 0.2560, *p* = 0.7761] nor an exposure condition × lever interaction [*F*_(2, 26)_ = 0.2083, *p* = 0.8133] were observed. These findings show that female mice are more motivated for sucrose reward on the RI lever, regardless of ethanol exposure condition.

### Effects of Ethanol on Progressive Ratio Microstructure

**FIGURE 4:**
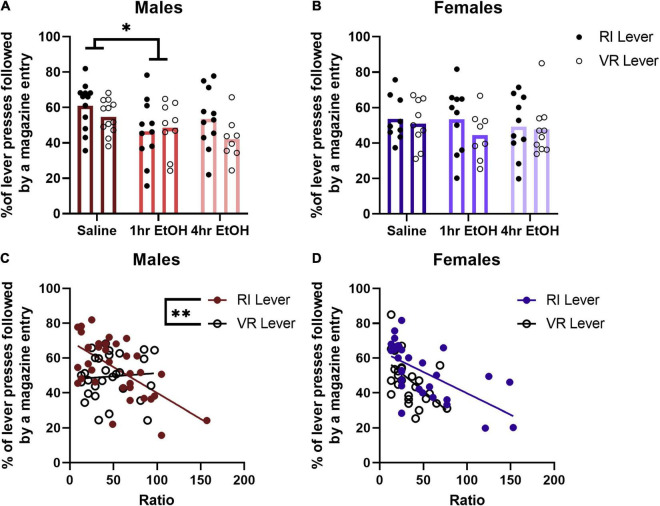
Magazine checking behavior after a lever press is related to performance on the PR. **(A)** One hour EtOH male mice check the magazine after lever pressing significantly less than saline mice. **(B)** There are no differences in magazine checking after lever pressing in females. **(C)** Magazine checking is correlated with performance only on the RI lever in males. **(D)** Whereas in females, magazine checking is correlated with PR performance on both levers. Data shown as mean ± SEM (*p < 0.05, **p < 0.01).

Reward magazine checking behavior was analyzed by comparing the percent of lever presses that were followed by a magazine entry for each PR session. In males (Figure 4A), a main effect of condition was observed [rmANOVA, *F*_(2, 57)_ = 3.742, *p* = 0.0297], with *post-hoc* analyses revealing that 1 h EtOH mice check the magazine after lever pressing significantly less than saline mice (Dunnett’s, *p* = 0.0383). There was no significant difference observed between 4 h EtOH and saline mice, although there was a trend toward reduced magazine checking after a lever press in the 4 h EtOH group vs. saline (Dunnett’s, *p* = 0.0519). This was not matched with differences in total magazine entries on the PR, as a rmANOVA analysis showed there were no significant main effects [exposure condition, *F*_(2, 32)_ = 1.981, *p* = 0.1545; lever, *F*_(1, 32)_ = 2.111, *p* = 0.1560] or interactions [exposure condition × lever, *F*_(2, 32)_ = 2.311, *p* = 0.1154] present. In females (Figure 4B), there were no significant main effects [rmANOVA, lever, *F*_(1, 24)_ = 3.090, *p* = 0.0915] or interactions [lever × exposure condition, *F*_(2, 24)_ = 0.9416, *p* = 0.4039] observed for magazine checking after a lever press. Similar to males, there were no differences in total magazine entries during PR testing observed in females [exposure condition, *F*_(2, 26)_ = 2.466, *p* = 0.1046; lever, *F*_(1, 26)_ = 3.104, *p* = 0.0899; exposure condition × lever, *F*_(2, 26)_ = 0.2042, *p* = 0.8166].

To determine whether magazine checking behavior after a lever press during the PR was related to the maximum ratio reached, a linear regression and correlational analysis was performed. No differences were found based on ethanol exposure for either males or females, so data were collapsed across exposure condition for each sex. For male mice (Figure 4C), there was a significant negative correlation between the ratio reached and magazine checking on the RI lever [*R*^2^ = 0.3781, *F*_(1, 32)_ = 19.46, *p* = 0.0001] but not VR lever [*R*^2^ = 0.0053, *F*_(1, 27)_ = 0.1432, *p* = 0.7081]. The slopes of the regression lines for the RI and VR lever in males were significantly different [*F*_(1, 59)_ = 8.044, *p* = 0.0062]. For female mice (Figure 4D), there was a significant negative correlation between the ratio reached and magazine checking observed on both the RI [*R*^2^ = 0.3906, *F*_(1, 27)_ = 17.31, *p* = 0.0003] and VR [*R*^2^ = 0.2953, *F*_(1, 25)_ = 10.48, *p* = 0.0034] levers. The slopes of these regression lines were not significantly different in females [*F*_(1, 52)_ = 1.319, *p* = 0.2560]. These findings suggest that magazine checking behavior after a lever press is related to reward motivation on the PR.

## Discussion

Our findings in males show that low dose ethanol exposure can drive sucrose reward motivation as only the males exposed to ethanol proximal to learning (1 h EtOH group), but not distal (4 h group) exhibited increased breakpoints on the PR. Moreover, the fact that this is due to a history of low dose ethanol and not acute exposure surrounding testing highlights a long-lasting outcome of low dose ethanol exposure. This increase in reward motivation in males was matched with reduced checking of the magazine after a lever press, suggesting that low dose ethanol may be shifting reward encoding and behavioral strategy in males. Furthermore, that there was a relationship between checking after a lever press with breakpoints only on the RI lever, not VR lever, suggests that males may be using strategies specific to reinforcement schedule history. In contrast to these findings, low-dose ethanol exposure did not impact sucrose reward motivation in female mice. There was an effect of reinforcement schedule history as females reached higher breakpoints on the RI lever as compared to the VR lever. However, since there was also an effect of schedule on response rate and reinforcers earned during training in females, it is difficult to separate the contributions of reinforcement schedule history alone on breakpoints in females.

Increased sucrose reward motivation was observed on the RI lever as compared to the VR lever in both males and females. RI schedules promote inflexible, habitual behavior that is insensitive to changes in the action-outcome relationship and to changes in reward value (Adams and Dickinson, 1981; Dickinson et al., 1983). It has long been theorized that habit and motivation are two separate processes (Dickinson et al., 2002; Everitt and Robbins, 2016), so the fact that there are schedule-dependent effects on reward motivation was unanticipated. The progressively increasing ratio of responding required to receive reinforcer delivery could be seen as a change in the action-outcome relationship. Insensitivity to this change as a result of overtraining on a RI schedule could then drive lever pressing on the PR. Thus, action-outcome insensitivity may be disguised as higher motivation on this test. Indeed, others have reported that mice which were insensitive to changes in outcome value also reached higher breakpoints on the PR, further supporting this interpretation (Gourley et al., 2016).

It may alternatively be that behavioral strategy is being shifted, as our male 1 h EtOH mice check the reward magazine less than saline mice regardless of lever. Our findings show that magazine checking behavior after a lever press and motivation as measured by a PR test are clearly related, but whether magazine checking just reflects motivational state or drives it is unknown. Magazine checking may reflect tracking and expectation of reward delivery, so mice that reach higher breakpoints thus exhibit less checking behavior because of high lever press-to-reward delivery ratios. Alternatively, greater magazine checking may reflect greater tracking of the outcome, or a greater attribution of value to the outcome.

This study showed that low dose ethanol can drive sucrose reward motivation in male mice, but whether it can drive ethanol reward motivation as well has yet to be determined. Some studies have shown that the effects of chronic ethanol on reward seeking behavior are similar for non-drug rewards and drug rewards (Kampov-Polevoy and Garbutt, 1997; Krahn et al., 2006; Sjoerds et al., 2013; Giacometti et al., 2020), whereas others find that they are different (Busse et al., 2005; Tryhus et al., 2021). One potential explanation for our findings is that low dose ethanol exposure after learning is shifting the way reward is encoded, thereby enhancing reward value, and increasing motivation. If this is the case, repeated low dose ethanol exposure is likely to increase reward motivation for an ethanol reward similarly to a sucrose reward. On the other hand, low dose ethanol exposure may interfere with sucrose metabolism and enhance reward value through this mechanism. If this is the case, these observed effects of low dose ethanol on reward motivation may not transfer to an ethanol reward as readily.

While there were no differences in responding or reinforcer delivery during training on the RI vs. VR lever in males, there were in females. In recent years, a number of tasks have been developed in which responding is maintained on multiple reinforcement schedules (Gremel and Costa, 2013; Barker et al., 2017b). The current findings indicate that under conditions in which schedules have been calibrated to match response rates in males, female mice discriminate between reinforcement schedules with a preference for responding on the interval schedule over the ratio schedule. The unmatched responding and subsequent reinforcer delivery in the females during training may relate to the schedule difference observed in PR testing. However, the presence of a main effect of reinforcement schedule in the males, where response rates were matched, suggests that these differences do not relate entirely to responding during training. Females respond differently to some stimuli then males, and behavioral measures that have been extensively used in males are not always appropriate, representative, or accurate measures in females (Chen et al., 2021; Shansky and Murphy, 2021). These results suggest that females are less inclined to respond on ratio schedules than interval schedules under conditions that are matched for males, and it will be important to consider this difference when designing experiments and matching schedules of reinforcement in the future. Additionally, it is possible that learning on two levers would yield different outcomes as compared to one lever or schedule, but our data and others do not suggest generalization of response strategies (Gremel and Costa, 2013; Barker et al., 2017b).

There is a paucity of research investigating the long-term impacts of low dose ethanol, especially in the context of reward, and this is particularly pronounced in female subjects. Women display shorter reaction times and greater cognitive performance following low to moderate alcohol consumption than men (Taberner, 1980; Dufouil et al., 1997), and have worse health outcomes at lower doses of ethanol than men (Foster et al., 2018). Thus, low dose ethanol appears to affect females differently than males, but the mechanisms underlying this are unknown. Physiologically, it has been shown that female rats exhibit greater accumbal dopamine levels following low dose ethanol exposure than males (Blanchard and Glick, 2002). So, it is possible that the dose used in these studies (0.5 g/kg) produces different physiological and behavioral effects in female mice than in males, and therefore does not increase sucrose reward motivation as observed in males.

This study was focused on determining the effects of low dose ethanol exposure and reinforcement schedule history on reward seeking in both males and females, but did not directly compare basal differences in task acquisition or progressive ratio responding in these groups (Garcia-Sifuentes and Maney, 2021). Future studies should be designed to investigate and compare different ethanol doses in males and females directly. There have been sex differences observed on the effects of ethanol on locomotor response in rodents, but this appears to depend heavily on the strain and species (Frye and Breese, 1981; Erickson and Kochhar, 1985; Middaugh et al., 1992). Sex differences have also been observed in sucrose reward seeking with higher doses of ethanol (Barker et al., 2017a), so it is possible that the ability of ethanol to modulate appetitive behavior is different between males and females across ranges of ethanol doses.

Multiple brain regions known to be important for the encoding and updating of reward value information are impacted by chronic ethanol (Lescaudron and Verna, 1985; DePoy et al., 2013; Barker et al., 2015; Trantham-Davidson et al., 2017; Ewin et al., 2019), and may be targets for the low dose ethanol effects observed here. In particular, the infralimbic prefrontal cortex, nucleus accumbens shell, and dentate gyrus have been shown to be activated as a result of low dose ethanol using c-Fos and Arc as markers of neuronal activity in rats, and this was not impacted by sex (Randall et al., 2020). Additionally, it has been shown that brain-derived neurotrophic factor (BDNF) is increased in the hippocampus following low to moderate alcohol consumption (Tizabi et al., 2018).

The 1–3 h window after learning encompasses protein synthesis dependent memory consolidation (Bourtchouladze et al., 1998) and is the primary time point where differences are observed in male mice here. Ethanol exposure during this critical period could impact normal protein synthesis associated with consolidation and may thus be enhancing reward motivation by shifting the way reward learning is encoded during training. This is further supported by the absence of an effect of ethanol administration 4 h after training on motivation, whereas ethanol exposure 1 h after training, during the period where protein synthesis-dependent consolidation/reconsolidation takes place, increased reward motivation in male mice. As the hippocampus is a region critical for memory consolidation and reconsolidation (Fanselow and Dong, 2010), changes in the activity of or protein synthesis in this region as a result of repeated low dose ethanol exposure could also be related to these observed differences, particularly in males.

This study demonstrated that repeated low dose ethanol exposure can enhance appetitive reward motivation, and that these effects were long lasting. These experiments advance our understanding of low dose ethanol exposure impact on maladaptive behavioral patterns that may contribute to aberrant drug and reward seeking, and further how sex may mediate this susceptibility.

## Data Availability Statement

The raw data supporting the conclusions of this article will be made available by the authors, without undue reservation.

## Ethics Statement

The animal study was reviewed and approved by Drexel University Institutional Animal Care and Use Committee.

## Author Contributions

KB designed the experiments together with JB. KB performed the experiments, analyzed the data, and wrote the manuscript with edits and input from JB. BS analyzed data for the experiments. All authors contributed to the article and approved the submitted version.

## Conflict of Interest

The authors declare that the research was conducted in the absence of any commercial or financial relationships that could be construed as a potential conflict of interest.

## Publisher’s Note

All claims expressed in this article are solely those of the authors and do not necessarily represent those of their affiliated organizations, or those of the publisher, the editors and the reviewers. Any product that may be evaluated in this article, or claim that may be made by its manufacturer, is not guaranteed or endorsed by the publisher.
